# 18F-Fluorodeoxyglucose positron emission tomography computed tomography detection of single organ vasculitis of the breast: A case report

**DOI:** 10.1097/MD.0000000000025259

**Published:** 2021-03-26

**Authors:** Taku Harada, Yosuke Sasaki, Takahiro Tokunaga, Ayuha Yoshizawa, Sakiko Miura, Keiichiro Ikeda, Tsukasa Saito, Juichi Hiroshige

**Affiliations:** a Division of General Medicine, Showa University Koto Toyosu Hospital, Tokyo; b Division of Diagnostic and Generalist Medicine, Dokkyo Medical University Hospital, Tochigi; c Department of General Medicine and Emergency Care, Toho University School of Medicine; d Department of Rheumatology; e Department of Breast Surgery, Showa University Koto Toyosu Hospital; f Department of Diagnostic Pathology, NTT Medical Center; g Department of Pathology, Showa University School of Medicine, Tokyo; h Department of Internal Medicine, Saitama Shinrin Hospital, Saitama, Japan.

**Keywords:** 18F-fluorodeoxyglucose positron emission tomography computed tomography, case report, fever of unknown origin, polyarteritis, single organ vasculitis

## Abstract

**Rationale:**

Although single organ vasculitis (SOV) is a rare occurrence and it is difficult to diagnose, its possibility as a cause of fever of unknown origin (FUO) must be considered. Recently, the usefulness of 18F-fluorodeoxyglucose positron emission tomography computed tomography (FDG PET/CT) in the diagnosis of unknown fevers due to vasculitis, especially in cases of small and medium-sized vasculitis, has begun to be pointed out.

**Patient concerns:**

We report the case of an 84-year-old woman with persisting fever for more than 2 weeks. She had no accompanying symptoms, other than fever, and the physical examination, echocardiography, and contrast-enhanced CT did not reveal any diagnostic clue.

**Diagnoses:**

The FDG PET/CT revealed positive uptakes of FDG in the left breast, with a standardized uptake value (SUV) of 2.9. The biopsy specimen of the left breast lesion revealed rupture of the elastic plate and evidence of fibrinoid necrosis of arteries, leading to the diagnosis of polyarteritis (PAN). Further angiographic examination and additional imaging did not reveal the presence of other lesions. Therefore, the diagnosis was established as a PAN-SOV of the left breast.

**Interventions:**

This patient has improved with follow-up only.

**Outcomes:**

There has been no evidence of a relapse of PAN over a 5-year follow-up period.

**Lessons:**

SOV presenting with unspecific local symptoms is difficult to diagnose based on the medical history and clinical examination. Our findings show that early “Combination of PET-CT and biopsy” can be a powerful diagnostic tool in patients with FUO for whom diagnosis of the underlying cause is difficult despite appropriate clinical examination.

## 1 Introduction

Although single organ vasculitis (SOV) is a rare occurrence and it is difficult to diagnose,^[1]^ its possibility as a cause of fever of unknown origin (FUO) must be considered. Breast vasculitis is an SOV that can result from varying causes, including giant cell arteritis (GCA), polyarteritis nodosa (PAN), and granulomatosis with polyangiitis (GPA).^[2]^ Among these causes, PAN is the most difficult to diagnose due to the absence of specific biomarkers, such as anti-neutrophil cytoplasmic autoantibodies. Consequently, the diagnosis of PAN requires invasive procedures, such as biopsy of the symptomatic area, for a histological diagnosis. However, fluorodeoxyglucose (FDG) positron emission tomography/computed tomography (FDG PET/CT) has been shown to be useful for the diagnosis of GPA-associated vasculitis of large,^[3,4]^ medium,^[5–7]^ and small^[8,9]^ vessels. Herein, we report on a case of PAN-SOV in which the use of FDG PET/CT played a critical role in the diagnosis. Our case underlines the possible usefulness of early FDG PET/CT for the non-invasive diagnosis of FUO associated with SOV and PAN more specifically.

## 2 Case presentation

**Figure 1:**
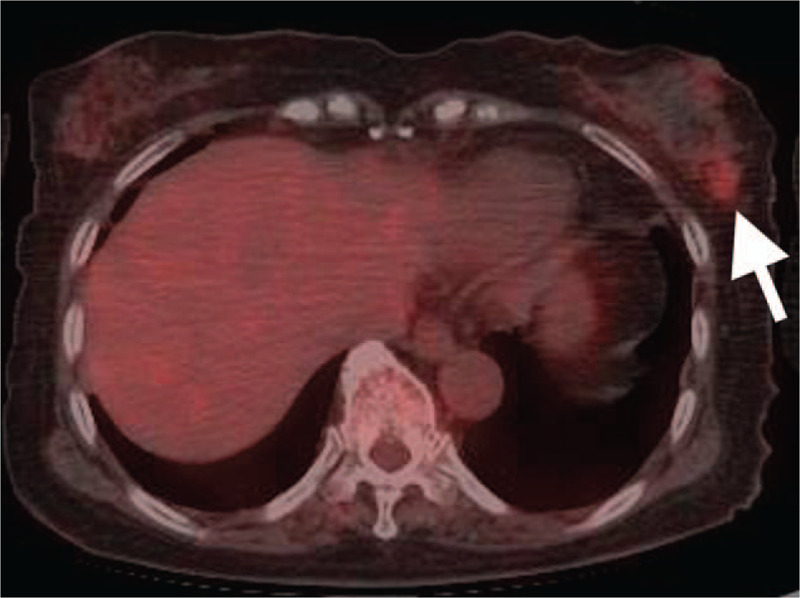
FDG PET/CT showing positive uptakes in the left breast (white arrowheads), with a standardized uptake value (SUV) of 2.9.

**Figure 2:**
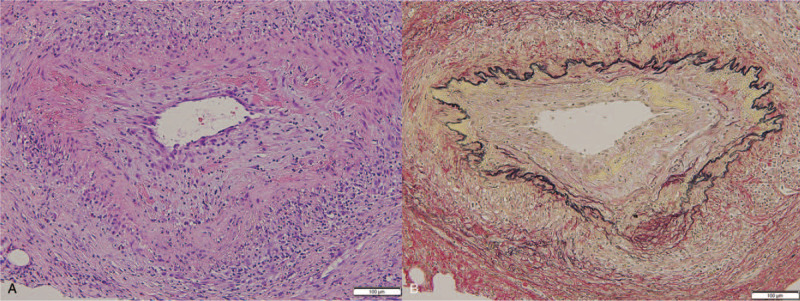
Histological findings of the left breast biopsy specimen. Hematoxylin and eosin staining (x 200) revealing a fibrinoid necrosis of the vessel wall, rupture of the internal elastic lamina, and presence of a mixed inflammatory infiltrate, composed of lymphocytes and plasma cells, extending into the surrounding medium-size vessels (A). Elastica Van Gieson stain (200×) revealing a rupture of the internal elastic lamina of medium-size vessels (B).

An 84-year-old woman was referred to our general medicine department for evaluation due to a history of persisting fever for more than 2 weeks. There were no accompanying symptoms, such as headache, chest pain, dyspnea, abdominal pain, back pain, diarrhea, constipation, arthralgia, rash, or numbness. Her general condition was good, with vital signs within normal range, with the exception of body temperature (38.2°C). The physical examination was unremarkable, with no evidence of joint inflammation or temporal arteritis. Positive laboratory results were identified with regard to leukocytosis (9300 /μl), elevation of serum C-reactive protein (181.2 mg/L), and the erythrocyte sedimentation rate (66 mm/hours). Liver and kidney function tests were normal, as were electrolyte levels. Antinuclear antibodies and anti-neutrophil cytoplasmic autoantibodies tests were also negative. Contrast-enhanced computed tomography (CT) imaging of the chest and abdomen and echocardiography were unremarkable. Additionally, 2 sets of blood cultures were negative. As examinations performed did not identify a possible cause for the FUO, we proceeded with FDG PET/CT to assess the possibility of a vasculitis or occult cancers. The FDG PET/CT revealed positive uptakes of FDG in the left breast, with a standardized uptake value (SUV) of 2.9 (Fig. 1). Based on the FDG PET/CT findings, we performed a biopsy of the left breast in the region of FDG uptake. The biopsy specimen revealed rupture of the elastic plate and evidence of fibrinoid necrosis of arteries. Based on these pathological findings, a diagnosis of PAN was made (Fig. 2). Further angiographic examination and additional imaging did not reveal the presence of other lesions. Therefore, the diagnosis was established as a PAN-SOV of the left breast. Although we did consider surgical excision of these lesions, the patient's symptoms and inflammatory response gradually improved, with the inflammatory response eventually subsiding. There has been no evidence of a relapse of PAN over a 5-year follow-up period.

## 3 Discussion

In this report, we describe a rare case of PAN localized to the left breast in an 84-year-old woman. Although rare, vasculitis confined to a single organ can affect any organ and/or area within an organ.^[1]^ Based on previous reports of vasculitis localized to the breast, >90% occur in women, at an average age of 61 years. In 70% of cases, symptoms are localized to the breast and include pain and tenderness, with no local symptoms being reported in the other 30% of cases. Systemic symptoms develop in 50% of cases and include the following: fever (35%), weight loss (15%), malaise or fatigue (21%), myalgia (26%), and arthralgia (26%).^[2]^ GCA, GPA, and PAN are the most frequently reported types of vasculitis involving the breast,^[2,10]^ with microscopic polyangiitis^[11]^ and Behcet's vasculitis also having been reported.^[12,13]^ In our case, the patient did not present with definite symptoms, other than fever and fatigue. Moreover, the lesion in the left breast could not be identified by careful and thorough physical examination or general imaging alone. However, FDG PET/CT was useful to identify regions of FDG uptake to guide biopsy, which confirmed the diagnosis.

As the detection of lesions ≤4 mm is not possible using FDG PET/CT, the diagnostic usefulness of FDG PET/CT for the diagnosis of angiitis has generally been considered to be limited to large-vessel involvement, such as GCA.^[14,15]^ However, in recent years, the effectiveness of FDG PET/CT for the diagnosis of small-vessel angiitis, mainly GPA, has been reported.^[8,9]^ In their case series of 16 patients, Soussan et al^[8]^ reported the successful detection of GPA using FDG PET/CT in all cases, including identification of a high FDG accumulation in the sinuses, lungs, cardiovascular organs, and kidneys. However, lesions in the skin, joints, eyes, and peripheral nerves were not detectable using FDG PET/CT.^[8]^ Kemna et al^[9]^ reported on the effectiveness of FDG PET/CT in detecting GPA lesions even when biochemical parameters are inconclusive and in the absence of local and clinical symptoms. Therefore, based on current evidence, FDG PET/CT is considered useful for the diagnosis of GPA.

Unlike GPA, the diagnostic accuracy of FDG PET/CT for PAN has not been extensively studied.^[16]^ Bleeker-Rovers et al^[5]^ reported on the effectiveness of FDG PET/CT in the evaluation of vasculitis, with a 100% positive predictive value and 82% negative predictive value. Two other studies reported on the accumulation of FDG detected by FDG PET/CT in cases of PAN.^[6,7]^ To the best of our knowledge, there has been no prior report on the use of FDG PET/CT for the diagnosis of Kawasaki disease, another type of vasculitis that affects medium-sized arteries. Additionally, the role of FDG PET/CT for the diagnosis of vasculitis of the central nervous system is also unclear.^[16]^

We should note that FDG/PET-CT itself, as an imaging tool, cannot differentiate SOV from other potential causes of FUO, such as infection, non-vasculitis related inflammatory disorders, or malignancy. Rather, FDG/PET-CT should be used to detect the target lesion for biopsy, which is especially useful when a lesion is undetectable by other imaging modalities, such as CT imaging.

Given the lack of evidence regarding the diagnostic effectiveness of FDG PET/CT for medium-sized vessel vasculitis, we believe that our experience demonstrates the possible role of “Combination of PET-CT and biopsy” for the diagnosis of PAN. Considering the difficulty in diagnosing PAN and its potential fatal outcome, FDG PET/CT may contribute to the timely diagnosis of PAN, allowing for early biopsy and treatment.

## 4 Conclusion

SOV presenting with unspecific local symptoms is difficult to diagnose based on the medical history and clinical examination. Our case report indicates that early “Combination of PET-CT and biopsy” can be a powerful diagnostic tool in patients with FUO for whom diagnosis of the underlying cause is difficult despite appropriate clinical examination.

## Acknowledgments

We thank Editage (www.editage.jp) for English-language editing.

## Author contributions

**Conceptualization:** Taku Harada, Yosuke Sasaki, Takahiro Tokunaga.

**Data curation:** Taku Harada, Takahiro Tokunaga, Ayuha Yoshizawa, Sakiko Miura, Keiichiro Ikeda.

**Investigation:** Taku Harada.

**Supervision:** Yosuke Sasaki, Takahiro Tokunaga, Ayuha Yoshizawa, Sakiko Miura, Keiichiro Ikeda, Tsukasa Saito, Juichi Hiroshige.

**Writing – original draft:** Taku Harada.

**Writing – review & editing:** Taku Harada, Yosuke Sasaki, Sakiko Miura.
